# Cost of Potentially Preventable Hospitalizations Among Adults With Chronic Kidney Disease: A Population-Based Cohort Study

**DOI:** 10.1177/20543581211018528

**Published:** 2021-06-04

**Authors:** Christy Chong, James Wick, Scott Klarenbach, Braden Manns, Brenda Hemmelgarn, Paul Ronksley

**Affiliations:** 1 Department of Community Health Sciences, Cumming School of Medicine, University of Calgary, AB, Canada; 2 Department of Medicine, Cumming School of Medicine, University of Calgary, AB, Canada; 3 Faculty of Medicine and Dentistry, University of Alberta, Edmonton, Canada

**Keywords:** administrative data, ambulatory care sensitive conditions, chronic kidney disease, preventable hospitalization, health care spending

## Abstract

**Background:**

Prior studies report high hospitalization rates among patients with chronic kidney disease (CKD) and approximately 10% to 20.9% of hospitalizations are potentially preventable.

**Objective:**

To determine the rate, proportion, and cost of potentially preventable hospitalizations and whether this varied by CKD category.

**Design:**

Retrospective cohort study using population-based data.

**Setting:**

Alberta, Canada.

**Patients:**

All adults with an outpatient serum creatinine measurement between January 1 and December 31, 2017 in the Alberta Kidney Disease Network data repository.

**Measurements:**

CKD risk categories were based on measures of proteinuria (where available), eGFR, and use of dialysis. Patients were linked to administrative data to capture frequency and cost of hospital encounters and followed until death or end of study (December 31, 2018). The outcomes of interest were the rate and cost of potentially preventable hospitalizations, as identified using the Canadian Institute for Health Information (CIHI)-defined ambulatory care sensitive condition (ACSC) algorithm and a CKD-related ACSC algorithm.

**Methods:**

Unadjusted and adjusted rates per 1000-patient years, proportions, and cost attributable to preventable hospitalizations were identified for the cohort as a whole and for patients within each CKD risk category.

**Results:**

Of the 1,110,895 adults with eGFR and proteinuria measurements, 181,422 had CKD. During a median follow-up of 1 year, there were 62,023 hospitalizations among patients with CKD resulting in a total cost of $946 million CAD; 6907 (11.1%) of these hospitalizations were for CIHI-defined ACSCs while 4323 (7.0%) were for CKD-related ACSCs. Adjusted rates of hospitalization for ACSCs increased with CKD risk category and were highest among patients treated with dialysis. Among CKD patients, the total cost of potentially preventable hospitalizations was $79 million and $58 million CAD for CIHI-defined and CKD-related ACSCs (8.4% and 6.2% of total hospitalization cost, respectively).

**Limitations:**

Based on the ACSC construct, we were unable to determine if these hospitalizations were truly preventable.

**Conclusions:**

Potentially preventable hospitalizations have a substantial cost and burden on the health care system among people with CKD. Effective strategies that reduce preventable admissions among CKD patients may lead to significant cost savings.

**Trial registration:**

Not applicable—observational study design

## Abrégé

**Contexte:**

Des études antérieures font état de taux élevés d’hospitalization chez les patients atteints d’insuffisance rénale chronique (IRC). Environ 10% à 20,9 % de ces hospitalisations seraient évitables.

**Objectifs:**

Établir l’incidence et la proportion des hospitalisations potentiellement évitables, de même que les coûts qui y sont attribuables, et vérifier si cela varie en fonction de la catégorie d’IRC.

**Type d’étude:**

Étude de cohorte rétrospective menée à partir de données basées sur une population.

**Sujets:**

Tous les adultes pour qui le référentiel de données du Alberta Kidney Disease Network disposait d’une mesure de créatinine sérique en consultation externe entre le 1er janvier et le 31 décembre 2017.

**Mesures:**

La mesure de protéinurie (lorsque disponible), le DFGe et la dialyze ont servi à établir les catégories de risque d’IRC. Les patients ont été couplés aux données administratives afin de saisir la fréquence des consultations à l’hôpital et le coût associé à celles-ci. Le suivi s’est poursuivi jusqu’au décès du patient ou jusqu’au 31 décembre 2018 (fin de l’étude). Les critères d’intérêt étaient l’incidence des hospitalisations évitables, de même que les coûts qui y sont attribuables; hospitalisations définies par l’algorithme de l’Institut canadien d’information sur la santé (ICIS) pour les conditions propices aux soins ambulatoires (CPSA) et par un algorithme des CPSA liées à l’IRC.

**Méthodologie:**

Les taux corrigés ou non pour 1000 années-patients et les proportions des hospitalisations évitables, de même que les coûts qui y sont attribuables, ont été déterminés pour l’ensemble de la cohorte et pour les patients de chaque catégorie de risque d’IRC.

**Résultats:**

Parmi les 1 110 895 adultes disposant de mesures de protéinurie et de DFGe, 181 422 étaient atteints d’IRC. Au cours d’un suivi médian d’un an, on a répertorié 62 023 hospitalisations de patients atteints d’IRC, ce qui représente un coût total de 946 millions de dollars canadiens. De ces hospitalisations, 6 907 (11,1 %) concernaient des CPSA définies par l’ICIS et 4 323 (7,0 %) concernaient des CPSA liées à l’IRC. Les taux corrigés d’hospitalization pour les CPSA augmentaient selon la catégorie de risque d’IRC et étaient plus élevés chez les patients dialysés. Chez les patients atteints d’IRC, le coût total des hospitalisations évitables s’établissait à 79 millions de dollars pour les CPSA définies par l’ICIS et à 58 millions de dollars canadiens pour les CPSA liées à l’IRC (respectivement 8,4 % et 6,2 % du coût total attribuable aux hospitalisations).

**Limites:**

La structure des CPSA ne nous a pas permis de déterminer si ces hospitalisations étaient effectivement évitables.

**Conclusion:**

Les hospitalisations évitables chez les patients atteints d’IRC représentent un fardeau et des coûts considérables pour le système de santé. Des stratégies efficaces qui permettraient de les réduire chez les patients atteints d’IRC entraîneraient des économies substantielles.

## Introduction

Chronic kidney disease (CKD) affects approximately 13% of the adult population^1^ and is associated with increased morbidity and mortality.^2,3^ Additionally, patients living with CKD often manage several comorbidities and may require acute care services for common complications.^4,5^ Among people living with CKD, high rates of hospitalization and substantial health care costs have been observed.^6-8^ Given the rising prevalence of CKD and associated health care expenditures,^9^ strategies are needed to reduce costs and improve the quality of care for patients with CKD.

Effective outpatient management of chronic conditions (including CKD) has been associated with a reduced risk of hospitalization, emergency department visits, and health care costs.^10-13^ A commonly used indicator to measure the adequacy of outpatient care is the identification of potentially preventable hospitalizations. Specifically, ambulatory care sensitive conditions (ACSCs) are defined as “medical conditions for which timely and effective outpatient care can help to reduce the risk of hospitalization by either preventing the onset of an illness, controlling an acute episodic illness, or management of a chronic disease.”^14^ There are specific ACSCs that are common among patients with CKD including volume overload, hyperkalemia, malignant hypertension, heart failure, diabetes with ketoacidosis, and diabetes with hyperosmolarity.^15^ While prior work has shown that strategies aimed at promoting timely and effective outpatient care, including the use of guideline-concordant medications, timely referral to a nephrologist, and routine monitoring and testing for albuminuria may reduce the risk for acute care use in patients with CKD,^16-18^ it is estimated that approximately 10% to 20.9% of patients with CKD will be hospitalized for a potentially preventable CKD-related ACSC.^19-21^ The characteristics of those at greatest risk of a CKD-related ACSC include elderly patients and those with specific comorbid conditions (diabetes, chronic liver disease, or heart failure).^20^ However, the health care costs attributable to these potentially preventable hospitalizations have not been quantified.

Given the known impact of CKD on health care costs,^9^ a greater understanding of the costs attributable to potentially preventable hospitalizations is required. In addition to quantifying the burden of preventable spending, this will inform whether targeted interventions could potentially reduce future health spending. Using a large population-based cohort, we aimed to quantify the rate, proportion, and cost of potentially preventable hospitalizations among patients with CKD and determine whether this varied by CKD risk category.

## Methods

### Data Source, Setting, and Study Population

We used a previously described provincial administrative health and laboratory data repository—the Alberta Kidney Disease Network.^22^ This is an established computerized repository of health data across Alberta that includes over 4.5 million adults.^22^ We created a cohort of adults (18 years of age and older) with one or more outpatient serum creatinine and proteinuria measurements between January 1, 2017 and December 31, 2017 in Alberta, Canada. We used measures of estimated glomerular filtration rate (eGFR) and proteinuria (when available) to determine kidney function. We then categorized patients into 6 risk categories (including patients treated with dialysis) based on the Kidney Disease Improving Global Outcomes (KDIGO) guidelines.^23^ This included: low risk (G1A1, and G2A1), moderate risk (G1A2, G2A2, and G3aA1), high risk (G1A3, G2A3, G3aA2, G3bA1), very high risk (G3aA3, G3bA2, G3bA3, G4A1, G4A2, G4A3, G5-NDA1, G5-NDA2, G5-NDA3, G4, G5-ND), dialysis (G5-DA1, G5-DA2, G5-DA3, G5-D), and high risk with unmeasured proteinuria (G3a and G3b). Individuals with CKD were included in the final cohort if they had: (a) an eGFR measurement ≥60 mL/min/1.73 m^2^ with measured proteinuria or (b) a series of 2 or more eGFR measurements <60 mL/min/1.73 m^2^ spanning 90 or more days (with or without a proteinuria measurement). The index eGFR was defined by the first eGFR measurement <60 mL/min/1.73 m^2^. Dialysis dependence was identified from the provincial dialysis registry.^24^ We excluded patients with only one eGFR <60 mL/min/1.73 m^2^ and those with only an eGFR ≥60 mL/min/1.73 m^2^ and unmeasured proteinuria to reduce the risk of misclassifying them as low, moderate, or high-risk CKD. Those with a prior kidney transplant were also excluded.

### Outcome—Identification of Inpatient Hospitalization Rate and Cost

To capture the frequency and cost of hospital encounters, patients were linked to provincial administrative health data. Specifically, the Discharge Abstract Database was used to identify demographic, administrative, and clinical data for hospitalizations. All patients were followed from their index eGFR measurement (ie, cohort entry) until death or end of study (December 31, 2018). The frequency of hospitalizations was recorded, and these data were used to determine the rate of all-cause and potentially preventable hospitalizations (number of hospitalizations per 1000 person-days), overall and for each CKD risk category. Hospital costs for typical Alberta hospital encounters were estimated using the Canadian Institute for Health Information (CIHI) resource intensity weights multiplied by the average cost of an inpatient encounter in Alberta during 2017/2018.^25^ Resource intensity weights are commonly used in the Canadian system for estimating the relative cost of hospital resources consumed.^26^ They are assigned based on clinical and demographic characteristics of individuals (ie, age, health status, and discharge status). All costs were reported in 2017 Canadian dollars (CAD) using the health care consumer price index.^27^

Costs for potentially preventable hospitalizations were defined for ACSC. We used an established algorithm from the CIHI to define ACSCs. These included the following 7 conditions: grand mal and other epileptic convulsions, chronic obstructive pulmonary disease (COPD), asthma, diabetes, heart failure/pulmonary edema, hypertension, and angina.^28^ We also explored 6 CKD-related ACSCs which include diabetes with ketoacidosis, diabetes hyperosmolarity, volume overload, hyperkalemia, malignant hypertension, and heart failure.^15,20^ The CKD-related ACSCs were previously developed using a Delphi technique and have been commonly used in studies to identify ACSCs in patients with CKD.^15,20^ The most responsible diagnosis code (defined as the medical diagnosis responsible for the greatest portion of a patient’s length of stay) was used to determine the presence of an ACSC. Given that CIHI-defined and CKD-related ACSCs both include diabetes, heart failure, and malignant hypertension, these constructs of potentially preventable hospitalizations are not mutually exclusive.

### Measurement of Covariates

Demographic and clinical variables were defined from Alberta Health administrative data and included age, sex, location of residence, median neighborhood household income quintile, general practitioner attachment, and comorbid conditions. Validated coding algorithms were used to identify the presence of 28 comorbidities based on International Statistical Classification of Diseases and Health Related Problems, Ninth (ICD-9) and Tenth (ICD-10) revision codes.^29^ General practitioner attachment was defined as the proportion of outpatient primary care encounters made to a single provider (among those with at least 3 visits) in the 2 years prior to entering the study. The level of attachment was defined for all individuals as poor (0%-50%), moderate (50%-74%), or high (75%-100%) using the Usual Provider Continuity Index.^30^

### Analysis

Descriptive statistics (means, standard deviations [SD], percentages, 95% confidence intervals (CIs), and interquartile ranges (IQR)) were used to describe demographic and clinical characteristics of the study cohort, stratified by KDIGO CKD risk category. Inpatient encounter characteristics for all-cause hospitalizations were explored, overall and by risk category of kidney disease. This included the percentage of individuals with at least one hospitalization, total number of hospital encounters, number of hospital admissions per patient, total number of hospital days, cumulative length of stay, discharge disposition, and number of patients with 1 or more 30-day all-cause readmission. Initially, we calculated unadjusted rates of all-cause hospitalizations per 1000 person-years. Due to overdispersion of count data, negative binomial models were used. The rates of all-cause hospitalizations were adjusted for age, sex, location of residence, median neighborhood household income quintile, general practitioner attachment, and comorbidities. Rates were adjusted to the sample proportions of the demographic and clinical characteristics of the cohort and reported by CKD category.

We repeated the analysis for potentially preventable hospitalizations using the CIHI-defined and CKD-related ACSCs and reported overall and stratified estimates by risk category of kidney disease. The cost of all-cause, CIHI-defined ACSCs, and CKD-related ACSC hospitalizations were identified for each level of kidney disease risk category. We also identified the number and percentage of inpatient encounters for the individual conditions that comprise the CIHI-defined ACSCs and CKD-related ACSCs, stratified by risk category of kidney disease. Stata 16 was used for all analyses (Stata Corp., College Station, TX).^31^

We followed the reporting standards outlined in the Strengthening the Reporting of Observational Studies in Epidemiology (STROBE) guidelines.^32^ This project was approved by the University of Calgary Conjoint Health Research Ethics Boards.

## Results

### Baseline Characteristics

**Figure 1.**
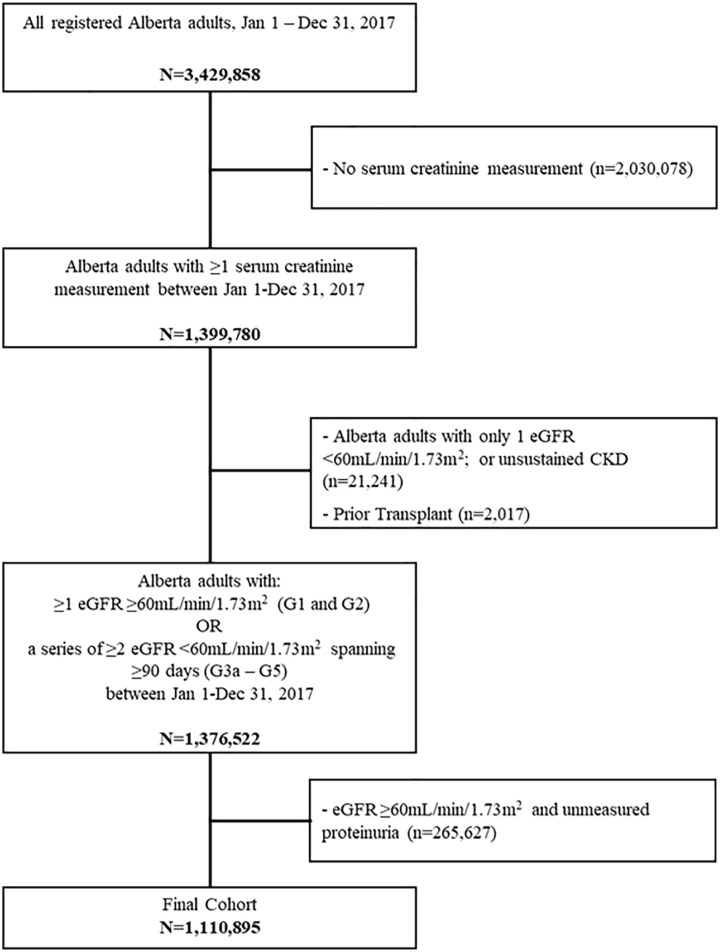
Study cohort formation. *Note.* eGFR = estimated glomerular filtration rate.

**Figure 2.**
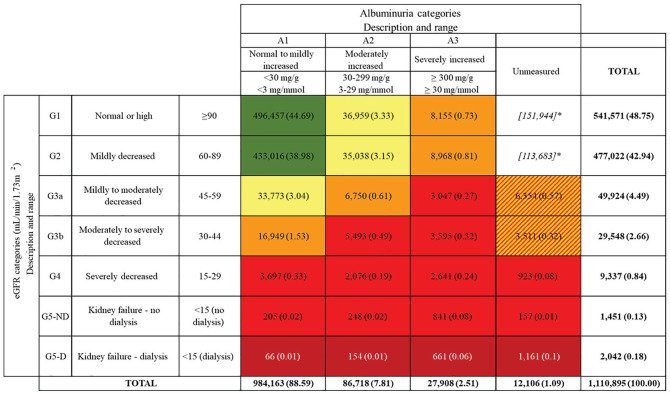
Risk category of kidney disease within study cohort based on kidney disease improving global outcomes chronic kidney disease criteria. *Note.* eGFR = estimated glomerular filtration rate.

Overall, 1,110,895 individuals aged 18 years and older who were residing in Alberta, Canada, with eGFR and proteinuria measurements between January 1, 2017 to December 31, 2017, were included in the final cohort (Figure 1). Of these, 181,422 individuals (16.3%) had CKD—of whom 58.3% were moderate risk, 22.5% were high risk, 5.4% were high risk with unmeasured proteinuria, 12.6% were very high risk, and 1.1% were dialysis-dependent (Figure 2). In the overall cohort, 54.6% were female and the mean age was 54.0 (SD: 16.9) years (Table 1). Hypertension was the most common comorbidity, ranging from 34.6% to 93.8% across CKD risk categories. A large proportion of individuals with CKD (moderate risk to dialysis-dependent) had 3 or more comorbidities.

**Table 1.** Demographic and Clinical Characteristics, Overall and by Risk Category of Kidney Disease.

Characteristic	Chronic kidney disease risk category						Overall (n = 1 110 895)
	Low (n = 929 473)	Moderate (n = 105 770)	High (n = 40 822)	Very High (n = 22 923)	Dialysis (n = 2042)	High with unmeasured proteinuria (n = 9865)	
Age, years							
Mean (SD)	51.8 (15.8)	61.4 (17.9)	68.1 (17.9)	75.0 (13.4)	63.9 (14.7)	79.5 (10.7)	54.0 (16.9)
18-44	34.6	20.2	13.1	3.4	11.4	0.4	31.4
45-64	44.0	32.3	22.6	16.6	38.4	9.4	41.2
65-74	14.7	22.6	22.0	24.0	25.2	21.7	16.0
75+	6.7	25.0	42.4	56.0	24.9	68.5	11.4
Sex, %							
Female	55.1	52.8	52.4	47.5	40.0	60.9	54.6
Male	44.9	47.2	47.6	52.5	60.0	39.1	45.4
Location of residence, %							
Rural	17.6	20.7	23	24.3	21.6	31.8	18.4
Urban	82.4	79.3	77	75.7	78.4	68.2	81.6
Median neighborhood household income quintile, %							
1 (lowest)	20.9	24.0	25.3	26.6	35.2	23.7	21.7
2	20.7	22.2	22.8	23.6	23.0	23.4	21.1
3	19.9	19.6	19.8	19.4	16.0	20.6	19.9
4	19.1	17.8	16.8	16.2	14.9	16.4	18.8
5 (highest)	19.2	16.5	15.2	14.1	10.8	15.9	18.7
General practitioner attachment, %							
<50%	24.1	22.4	22.4	22.3	35.7	20.0	23.8
50%-74%	33.6	32.0	31.2	31.3	32.2	30.8	33.3
75%-100%	42.2	45.7	46.4	46.4	32.1	49.2	42.9
Comorbidities, %							
Alcoholism	3.0	4.0	4.2	4.5	8.6	3.4	3.1
Asthma	2.5	3.6	4.3	5.1	7.0	4.7	2.8
Atrial fibrillation	2.8	8.1	14.6	21.2	17.1	22.4	4.3
Cancer (lymphoma)	0.4	0.8	1.1	1.7	2.7	1.6	0.5
Cancer (metastatic)	0.7	1.1	1.7	2.1	1.8	2.5	0.8
Cancer (non-metastatic)	3.3	5.1	6.7	7.7	6.4	8.9	3.7
Congestive heart failure (CHF)	2.3	8.0	17.1	31.0	46.5	25.6	4.2
Chronic pain	14.5	16.8	18.4	19.2	19.8	16.7	15.0
Chronic obstructive pulmonary disease (COPD)	9.9	17.4	23.7	31.3	31.1	28.8	11.7
Hepatitis B	0.4	0.3	0.2	0.3	0.9	0.1	0.4
Cirrhosis	0.2	0.5	0.8	1.1	2.5	1.0	0.3
Dementia	1.5	4.4	8.6	11.7	7.2	15.8	2.3
Depression	12.0	12.9	12.6	11.4	13.8	10.8	12.1
Diabetes	14.6	37.4	41.9	58.0	63.8	24.2	18.9
Epilepsy	2.0	2.5	2.7	2.8	6.4	2.8	2.1
Hypertension	34.6	64.2	77.9	93.2	93.8	83.1	40.7
Hypothyroidism	12.9	16.2	19.8	22.3	16.8	25.6	13.8
Inflammatory bowel disease	1.5	1.8	1.9	2.2	2.4	2.0	1.5
Irritable bowel syndrome	2.7	2.7	3.1	2.8	1.9	2.7	2.7
Multiple sclerosis	1.0	1.0	1.1	0.9	1.6	0.8	1.0
Myocardial infarction	1.8	4.6	7.5	11.4	12.4	9.5	2.6
Parkinson’s disease	0.5	1.1	1.9	2.0	1.4	2.3	0.7
Peptic ulcer disease	0.1	0.2	0.3	0.6	1.4	0.4	0.1
Peripheral vascular disease	0.9	2.5	4.6	8.0	30.5	5.6	1.4
Psoriasis	0.9	1.1	1.3	1.6	1.9	1.4	0.9
Rheumatoid arthritis	2.6	4.1	5.6	6.1	6.2	6.6	3.0
Schizophrenia	1.2	1.5	1.7	1.5	2.4	1.7	1.3
Stroke	5.2	11.3	17.4	24.8	26.0	23.4	6.8
Number of comorbidities, %							
0	34.7	15.0	8.8	2.1	2.6	5.1	30.8
1	28.5	20.7	14.6	8.3	6.8	13.9	26.7
2	18.3	23.9	21.5	18.1	12.3	19.6	19.0
3+	18.3	40.3	55.0	71.5	78.1	61.4	23.5

In the overall cohort, there were 186,759 all-cause hospitalizations; 62,023 hospitalizations occurred among those with CKD within the 1-year follow-up (Table 2). While 83.8% of all person-time occurred within the low-risk CKD group, adjusted rates of all-cause hospitalization increased with CKD risk category and was highest among patients treated with dialysis (560.0 [95% CI: 509.5-610.4] hospitalizations per 1000 person-years). The median cumulative length of stay over the entire follow-up period followed a similar pattern with low risk CKD patients staying in hospital on average for 4 days (IQR: 2-8 days) compared with very high risk (12 days [IQR: 6-32 days]) and patients treated with dialysis (15 days [IQR: 6-40 days]) staying for longer periods of time. Overall, total follow-up time was 1,103,938 person-years and 10,866 individuals (1%) died within the study follow-up period. Mortality was highest among patients treated with dialysis (11.2% of this risk group). Approximately 11.8% of individuals in the cohort had at least one 30-day all-cause readmission. This was also highest among the dialysis-dependent group (27.3% had at least one readmission).

**Table 2.** Hospital Encounter Characteristics for All-Cause Hospitalizations, Overall and by Kidney Disease Risk Category.

Variable	Chronic kidney disease risk category						Overall (n = 1 110 895)
	Low (n = 929 473)	Moderate (n = 105 770)	High (n = 40 822)	Very high (n = 22 923)	Dialysis (n = 2042)	High with unmeasured proteinuria (n = 9865)	
Total number of hospital encounters, n	124 736	26 453	15 056	14 303	2402	3809	186 759
Percent of all hospital encounters, %	66.8	14.2	8.1	7.7	1.3	2.0	100.0
Number of hospital admissions per patient, mean (95% CI)	0.134 (0.133-0.135)	0.250 (0.246-0.254)	0.369 (0.360-0.377)	0.624 (0.609-0.640)	1.178 (1.105-1.252)	0.387 (0.369-0.405)	0.168 (0.167-0.169)
Person-time, years	924,846	104,880	40,291	22,437	1948	9536	1,103,938
Percent of all person-time, %	83.8	9.5	3.6	2.0	0.2	0.9	100.0
Unadjusted hospitalization rate, per 1000 person-years (95% CI)	136.3 (135.4-137.2)	257.8 (253.4-262.2)	386.8 (377.1-396.5)	664.7 (644.4-685.0)	1290.2 (1167.3-1413.2)	414.5 (393.5-435.6)	172.5 (171.1-173.6)
Adjusted hospitalization rate^a^, per 1000 person-years (95% CI)	128.9 (127.9-129.9)	170.2 (167.0-173.4)	193.6 (188.4-198.8)	266.4 (258.0-274.8)	560.0 (509.5-610.4)	157.0 (148.6-165.4)	137.9 (136.9-138.9)
All-cause mortality, n (%)	5667 (0.6)	1793 (1.7)	1284 (3.2)	1357 (5.9)	227 (11.2)	538 (5.5)	10,866 (1.0)
Individuals with at least one hospitalization, n (%)	93,215 (10.0)	17,911 (16.9)	9165 (22.5)	7554 (33.0)	1079 (52.8)	2266 (23.0)	131,190 (11.8)
Total number of hospital days^b^, n	1,025,565	278,949	193,050	206,075	37,797	53,826	1,795,262
Percentage of total hospital days, %	57.1	15.5	10.8	11.5	2.1	3.0	100.0
Cumulative length of stay, median (IQR)^b^	4 (2-8)	5 (3-13)	8 (4-22)	12 (6-32)	15 (6-40)	10 (5-27)	4 (3-11)
Discharge Disposition (for all admissions), % (95% CI)^b^							
Transfer to acute care	1.9 (1.8-1.9)	2.9 (2.7-3.1)	3.9 (3.6-4.2)	5.1 (4.8-5.5)	6.0 (5.1-7.0)	5.1 (4.4-5.7)	2.4 (2.4-2.5)
Transfer to long-term care	1.4 (1.3-1.5)	2.7 (2.5-2.9)	5.1 (4.7-5.5)	6.1 (5.6-6.6)	6.3 (5.1-7.6)	8.4 (7.3-9.5)	2.3 (2.2-2.3)
Transfer to other facility	0.4 (0.4-0.5)	0.6 (0.5-0.7)	0.8 (0.7-1.0)	1.1 (0.9-1.3)	1.0 (0.6-1.4)	1.6 (1.2-2.1)	0.5 (0.5-0.6)
Home with support services	7.6 (7.4-7.7)	12.9 (12.5-13.4)	19.3 (18.6-20.1)	24.6 (23.8-25.5)	16.1 (14.2-18.0)	25.8 (24.1-27.5)	10.5 (10.3-10.6)
Home	86.2 (86.0-86.5)	77.4 (76.8-77.9)	65.7 (64.8-66.6)	56.8 (55.8-57.9)	59.4 (56.9-62.0)	52.2 (50.2-54.1)	81.2 (81.0-81.4)
Signed out against medical advice	0.7 (0.7-0.8)	0.7 (0.6-0.8)	0.8 (0.7-1.0)	0.6 (0.4-0.7)	2.4 (1.6-3.1)	0.4 (0.2-0.7)	0.7 (0.7-0.8)
In-hospital mortality	1.7 (1.7-1.8)	2.8 (2.6-3.0)	4.3 (3.9-4.7)	5.6 (5.2-6.1)	8.6 (7.2-10.1)	6.4 (5.4-7.3)	2.4 (2.3-2.5)
Number of patients with 1 or more 30-day all-cause readmissions^b^, n (%)	9230 (9.9)	2308 (12.9)	1593 (17.4)	1608 (21.3)	294 (27.3)	398 (17.6)	15431 (11.8)

*Note.* CI = confidence interval; IQR = interquartile range.

a Adjusted for age, sex, location of residence, median neighborhood household income quintile, general practitioner attachment, and comorbidities.

b Among individuals with at least one hospitalization in the follow-up period.

### Potentially Preventable Hospitalizations

Among those with moderate to dialysis-dependent CKD, the total number of hospitalizations for CIHI-defined and CKD-related ACSCs were 6907 (11.1%) and 4323 (7.0%), respectively (Table 3). As CKD risk category increased, the unadjusted and adjusted hospitalization rates for CIHI-defined ACSCs increased. A similar trend for unadjusted and adjusted hospitalization rates for CKD-related ACSCs was observed. Forty-two percent of CIHI-defined ACSC encounters and 26.2% of CKD-related ACSC encounters occurred in the low-risk CKD group. The highest adjusted hospitalization rates for CIHI-defined and CKD-related ACSCS were among patients who were dialysis-dependent (14.4 [95% CI: 9.8-19.0] and 12.7 (95% CI: 9.5-15.9) hospitalizations per 1000 person-years, respectively). The largest number of CIHI-defined ACSC encounters were for COPD (37.7%) (Online Appendix 1). Conversely, most of the encounters for CKD-related ACSCs were due to heart failure across all CKD risk categories (82.5%).

**Table 3.** Hospital Encounters, Person-Time, and Hospitalization Rates for CIHI-Defined ACSC and CKD-Related ACSC.

	Variable	Chronic kidney disease risk category						Overall (n = 1 110 895)
		Low (n = 929 473)	Moderate (n = 105 770)	High (n = 40 822)	Very high (n = 22 923)	Dialysis (n = 2042)	High with unmeasured proteinuria (n = 9865)	
**CIHI-defined** **ACSC hospitalization**	Total number of hospital encounters, n	4993	2057	1744	2341	202	563	11 900
	Percent of all hospital encounters, %	42.0	17.3	14.7	19.7	1.7	4.7	100.0
	Person-time, years	924 846	104 880	40 291	22 437	1948	9536	1 103 938
	Percent of all person-time, %	83.8	9.5	3.6	2.0	0.2	0.9	100.0
	Unadjusted hospitalization rate, per 1000 person-years (95% CI)	4.1 (3.9-4.2)	13.9 (12.8-14.9)	28.9 (25.5-32.3)	86.7 (73.4-100.1)	112.0 (68.6-155.5)	43.1 (29.8-56.4)	6.6 (6.4-6.9)
	Adjusted hospitalization rate^a^, per 1000 person-years (95% CI)	2.4 (2.2-2.5)	3.9 (3.5-4.3)	5.3 (4.6-6.0)	9.9 (8.4-11.3)	14.4 (9.8-19.0)	5.7 (3.9-7.5)	2.6 (2.4-2.7)
**CKD-related** **ACSC hospitalization**	Total number of hospital encounters, n	1537	994	1049	1734	175	371	5860
	Percent of all hospital encounters, %	26.2	17.0	17.9	29.6	3.0	6.3	100.0
	Person-time, years	924 846	104 880	40 291	22 437	1948	9536	1 103 938
	Percent of all person-time, %	83.8	9.5	3.6	2.0	0.2	0.9	100.0
	Unadjusted hospitalization rate, per 1000 person-years (95% CI)	4.3 (4.1-4.4)	17.3 (16.3-18.3)	41.7 (38.7-44.6)	106.2 (97.6-114.8)	121.1 (88.6-153.6)	63.9 (55.3-72.6)	9.7 (9.4-0.0)
	Adjusted hospitalization rate^a^, per 1000 person-years (95% CI)	2.3 (2.2-2.5)	3.6 (3.3-3.9)	4.7 (4.3-5.2)	8.1 (7.3-8.9)	12.7 (9.5-15.9)	4.9 (4.2-5.6)	2.6 (2.5-2.8)

*Note.* CIHI = Canadian Institute for Health Information; ACSC = ambulatory care sensitive condition; CKD = chronic kidney disease; CI = confidence interval.

a Adjusted for age, sex, location of residence, median neighborhood household income quintile, general practitioner attachment, and comorbidities.

### All-Cause Hospitalization Costs

**Figure 3.**
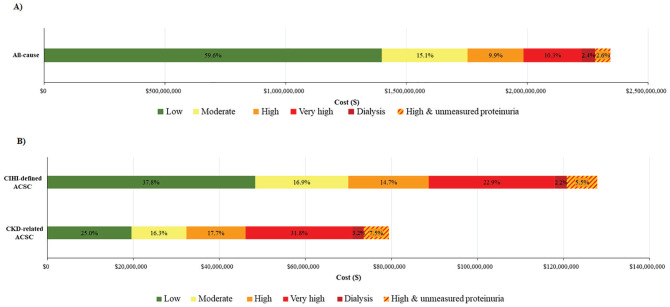
Proportion of all-cause (Panel A) and CIHI-defined ACSC or CKD-related ACSC (Panel B) hospital costs attributable to each level of kidney disease risk category. *Note*. CIHI = Canadian Institute for Health Information; ACSC = ambulatory care sensitive condition; CKD = chronic kidney disease.

Of the entire cohort, $2.3 billion CAD was spent on all-cause hospitalizations and the average cost of per patient (with at least one serum creatinine measurement) in Alberta was $11,334 (95% CI: $11,227-$11,441 CAD) (Figure 3; Table 4). Those with low-risk CKD had the highest total cost ($1.3 billion CAD) with an average cost per patient of $10,133 (95% CI: $10,020-$10,246). Among patients with CKD (moderate risk to dialysis-dependent), the total cost of all-cause hospitalizations was $946 million CAD. Patients treated with dialysis made up a small percentage of the cohort (0.18%) but accounted for a disproportionate amount of the spending ($56 million CAD) among CKD patients. They also had a significantly higher average all-cause hospitalization cost per patient ($24,970 CAD [95% CI: $22,561-$27,379 CAD]) compared to other CKD risk categories. Furthermore, the average cost of hospitalization per patient on dialysis was approximately 2.5 times those with low risk CKD.

**Table 4.** Cost of All-Cause, CIHI ACSC, and CKD-Related ACSC Hospitalizations by Kidney Disease Risk Category.

	Variable	Chronic Kidney Disease Risk Category						Overall (n = 131 190)
		Low (n = 93 215)	Moderate (n = 17 911)	High (n = 9165)	Very high (n = 7554)	Dialysis (n = 1079)	High with unmeasured proteinuria (n = 2266)	
**All-cause hospitalization**	Total number of hospital encounters, n	124 736	26 453	15 056	14 303	2402	3809	186 759
	Percent of total hospitalizations, %	66.8	14.2	8.1	7.7	1.3	2.0	100.0
	Total cost, $	$1 398 326 016	$354 497 824	$231 722 624	$241 157 280	$56 527 540	$62 098 184	$2 344 329 472
	Percent of total cost, %	59.6	15.1	9.9	10.3	2.4	2.6	100.0
	Cost, median (IQR)	$6,407.9 ($4180.1-$10 222.2)	$6,876.4 ($4235.2-$11 827.3)	$8,380.8 ($5054.7-$14 718.9)	$9,499.9 ($5781.7-$17 669.3)	$13,884.6 ($8826.4-$25 272.6)	$9,209.6 ($5591.4-$17 159.8)	$6,643.5 ($4207.8-$11 137.8)
	Cost, mean (95% CI)	$10 133.5 ($10 020.7-$10 246.4)	$12 267.8 ($11 962.7-$12 572.9)	$14,703.9 ($14 210.9-$15 196.9)	$16 629.1 ($16 036.3-$17 221.9)	$24 970.5 ($22 561.9-$27 379.1)	$15 597.2 ($14 548.5-$16 645.9)	$11 334.6 ($11 227.9-$11 441.3)
**CIHI-defined** **ACSC hospitalization**	Number of ACSC encounters, n	4993	2057	1744	2341	202	563	11 900
	Percent of total ACSC encounters, %	42.0	17.3	14.7	19.7	1.7	4.7	100.0
	Total ACSC cost, $ (%)	$48 388 760 (3.5)	$21 583 450 (6.1)	$18 765 752 (8.1)	$29 241 144 (12.1)	$2 793 587 (4.9)	$7 095 311 (11.4)	$127 868 000 (5.5)
	Percent of total ACSC cost, %	37.8	16.9	14.7	22.9	2.2	5.5	100.0
	Cost, median (IQR)	$5985.9 ($4784.7-$8923.8)	$6876.4 ($5054.7-$10 121.6)	$6959.2 ($5779.3-$10 402.8)	$8313.7 ($5779.3-$12 249.2)	$10 012.5 ($7177.9-$16 270.3)	$7562.1 ($5779.3-$12 255.1)	$6876.4 ($5054.7-$10 335.1)
	Cost, mean (95% CI)	$9701.2 ($9216.9-$10 185.4)	$10 544.9 ($9786.4-$11 303.4)	$10,719.1 ($9,994.6-$11,443.5)	$12 466.6 ($11 694.9-$13 238.3)	$14 282.8 ($11 779.8-$16 785.8)	$12 784.6 ($11 172.8-$14 396.5)	$10 717.3 ($10 400.2-$11 034.4)
**CKD-related** **ACSC hospitalization**	Number of ACSC encounters, n	1537	994	1049	1734	175	371	5860
	Percent of total ACSC encounters, %	26.2	17.0	17.9	29.6	3.0	6.3	100.0
	Total ACSC cost, $ (%)	$19 605 157 (1.4)	$12 744 452 (3.6)	$13 824 110 (6.0)	$24 893 287 (10.3)	$2 530 483 (4.5)	$5 855 185 (9.4)	$78 278 114 (3.3)
	Percent of total ACSC cost, %	25.0	16.3	17.7	31.8	3.2	7.5	100.0
	Cost, median (IQR)	$6876.4 ($5115.5-$11 027.5)	$7122.6 ($5779.3-$12 249.2)	$8055.9 ($5779.3-$12 249.2)	$8529.2 ($5848.8-$14 186.9)	$9569.5 ($6798.2-$16 270.3)	$8413.8 ($5779.3-$14 575.6)	$7593.6 ($5779.3-$12 249.2)
	Cost, mean (95% CI)	$12,755.5 ($11 380.3-$14 130.7)	$12 821.4 ($11 760.0-$13 882.7)	$13 178.4 ($11 488.1-$14 868.7)	$14 355.9 ($13 388.1-$15 323.8)	$14 459.9 ($12 109.8-$16 809.9)	$15 782.2 ($11 562.8-$20 001.5)	$13 358.0 ($12 662.9-$14 053.2)

*Note.* CIHI = Canadian Institute for Health Information; ACSC = ambulatory care sensitive condition; CKD = chronic kidney disease; CI = confidence interval; IQR = interquartile range.

### Costs for Potentially Preventable Hospitalizations

The total cost of CIHI-defined ACSC hospitalizations for the overall cohort was $127 million CAD with an average cost per patient of $10,717 CAD (95% CI: $10,400-$11,034 CAD) (Figure 3; Table 4). The low risk CKD group accounted for a total cost of $48 million CAD, whereas patients with CKD (moderate risk to dialysis-dependent) accounted for a majority of the cost at $79 million CAD. Patients who were dialysis-dependent had the highest average CIHI-defined ACSC cost ($14,282 CAD [95% CI: $11,779-$16,785 CAD]).

For CKD-related ACSC hospitalizations, the total cost was $78 million CAD in the overall cohort, with an average cost per encounter of $13,358 CAD (95% CI: $12,662-$14,053 CAD). Among patients with CKD (moderate risk to dialysis-dependent), $58 million CAD (6.2% of total hospitalization costs) was spent on CKD-related ACSC hospitalizations. Very high risk patients had the largest total CKD-related ACSC spending ($24 million CAD) which was approximately 10 times the total amount spent among patients treated with dialysis ($2.5 million CAD). Similar to the all-cause and CIHI-defined ACSC hospitalization spending for patients with CKD, the average CKD-related ACSC cost per hospital stay for an ACSC among patients treated with dialysis ($14,459 CAD [95% CI: $12,109-$16,809 CAD]) was higher than the average cost for patients with moderate ($12,821 CAD [95% CI: $11,760-$13,882 CAD]), high ($13,178 CAD [95% CI: $11,488-$14,868 CAD]), and very high (non-dialysis) ($14,355 CAD [95% CI: $13,388-$15,323 CAD]) CKD.

## Discussion

Within this population-based cohort we found that almost one-third of all-cause hospitalizations were among patients with CKD (moderate risk to dialysis-dependent) which contributed to a total annual hospitalization cost of $946 million CAD. Adjusted rates of all-cause and potentially preventable hospitalizations increased with CKD risk category, with patients treated with dialysis having a disproportionately higher average cost for all-cause hospitalizations per patient relative to other CKD risk categories. Furthermore, potentially preventable hospitalizations were common, with total hospital spending reaching $79 million CAD for CIHI-defined ACSCs and $58 million CAD for CKD-related ACSCs within a 1-year period. Among those with CKD, the average CIHI-defined and CKD-related ACSC costs per person were highest among patients treated with dialysis. These findings are important from a health management perspective as they suggest opportunities for cost savings exist by focusing on strategies that target patients with CKD.

Prior studies have focused on total health care spending among patients with CKD, including prescription medications, physician visits, emergency department visits, outpatient procedures, and hospitalizations.^6,7,9,33^ The mean unadjusted cumulative annual cost of care for patients with CKD (as defined by an eGFR <60 mL/min/1.73 m^2^ and excluding patients on dialysis or transplant recipients) has been estimated to be $14,634 CAD in Alberta, Canada.^9^ When extrapolated to Canada, approximately $32 billion CAD per year is spent on patients with CKD.^9^ However, given that hospital spending accounts for the largest component of total health care expenditures combined with the fact that hospitalization rates are high among patients with CKD,^6-8^ our work purposefully focused on spending within this healthcare sector. Moreover, a top research priority noted by patients with CKD is the need for hospitalization avoidance strategies.^34^ Our study contributes to this priority as we determined annual hospital spending, the proportion of hospital spending that was potentially preventable and whether this varied by CKD risk category.

To our knowledge, this study is the first to quantify all-cause hospitalizations and potentially preventable cost across CKD risk category at a population level. We found that those who are dialysis-dependent had the highest average all-cause hospitalization expenditures. This finding is consistent with the current literature on dialysis treatment being a key driver of cost in this patient population^7,35-37^. For instance, a study from Sweden found that the highest mean annual cost was observed among patients who were hemodialysis-dependent with costs 45-times higher than age-, sex- and index year- matched individuals from the general population.^7^ The total cost for patients on dialysis was also significantly higher than the cost for non-dialysis CKD and transplant patients in the United States.^33^ While these studies highlight the costs attributable to kidney failure, we extend this work by identifying where cost savings could be realized. Conversely, patients with low risk CKD accounted for the largest proportion of the total all-cause hospital expenditures despite having the lowest per-person costs. This suggests that upstream strategies targeting this large proportion of the population may also result in significant cost savings.

Relative to other CKD risk categories, we found that the average cost per patient for potentially preventable ACSC hospitalizations was highest among those that were dialysis-dependent. Notably, 82.5% of CKD-related ACSCs were for heart failure. It is well known that individuals with more advanced CKD (particularly those that are dialysis-dependent) are at greatest risk for developing heart failure^38-40^ and various strategies have been devised to address this comorbid condition. This includes enhancing methods to assess and control fluid overload using dietary and pharmacologic interventions, as well as dialysis treatments in relevant patients. The use of pharmacotherapy (eg, beta-blockers, angiotensin-converting enzyme inhibitors, or angiotensin receptor blockers) among patients with varying risk categories of CKD has been another strategy.^41^ The implementation of various heart failure intervention programs has been shown to reduce hospitalizations and improve outcomes.^42,43^ However, our findings suggest the need for the development of interventions that target underlying comorbidities, particularly heart failure, in earlier CKD risk patients before the onset of dialysis treatment. This strategy could improve patient outcomes and reduce potentially preventable hospitalization costs. Future research is required to explore additional reasons for preventable spending and whether interventions that target CKD care could improve quality of care and reduce spending.

Our study has a number of strengths, including the use of population-based data for all adults in Alberta. Contrary to previous CKD-related ACSC studies, we were also able to capture a larger proportion of hospitalizations that were potentially preventable by identifying CKD-related ACSCs and CIHI-defined ACSCs. However, this study should be interpreted in light of its limitations. First, we used the ACSC construct to quantify potentially preventable spending. It is well recognized that many patient, provider, and system-level factors influence how patients use the health care system. It is unclear if the hospitalization costs were completely attributable to the admitting diagnosis as it is possible for some complications to have occurred during hospitalization that were unrelated to the admitting diagnosis. Furthermore, our analysis did not include social factors such as community support, food security, or education that may differentially affect CKD risk categories and the rates of hospitalizations for ACSCs. While our estimates represent a spectrum of preventability that require an exploration into other aspects of patient care within the community, we were able to account for important factors related to income and primary care attachment within our adjusted analysis. Second, it is possible that our findings are not generalizable to other jurisdictions with variations in primary care delivery, capacity to care for patients within the community, and patient characteristics. However, given that we used a population-based dataset which contained laboratory measurements for all adults diagnosed with CKD to quantify hospital spending across CKD risk category, this issue may be mitigated.

## Conclusion

We found that hospital spending is high among patients with CKD and varies by disease risk category. While a relatively low proportion of total hospital spending is considered potentially preventable, these annual estimates ($79 million and $58 million CAD for CIHI-defined and CKD-related ACSC, respectively) are substantial and suggest the need to explore opportunities for cost savings by focusing on patients with CKD and the management of specific comorbid conditions (eg, heart failure). Although our study suggests that health care savings may be realized through targeted interventions aimed at CKD groups with specific comorbidity profiles, a greater understanding of the different social, behavioral, physician, and health system factors that contribute to the construct of potentially preventable hospitalizations are required. This represents an important area for future research that has the potential to improve overall patient care while addressing high inpatient spending within a growing high-risk patient population.

## Supplemental Material

sj-pdf-1-cjk-10.1177_20543581211018528 – Supplemental material for Cost of Potentially Preventable Hospitalizations Among Adults With Chronic Kidney Disease: A Population-Based Cohort Study Click here for additional data file. Supplemental material, sj-pdf-1-cjk-10.1177_20543581211018528 for Cost of Potentially Preventable Hospitalizations Among Adults With Chronic Kidney Disease: A Population-Based Cohort Study by Christy Chong, James Wick, Scott Klarenbach, Braden Manns, Brenda Hemmelgarn and Paul Ronksley in Canadian Journal of Kidney Health and Disease
