# Supplementary material for: Cost of Potentially Preventable Hospitalizations Among Adults With Chronic Kidney Disease: A Population-Based Cohort Study
Source: Can J Kidney Health Dis. 2021 Jun 4;8:20543581211018528. doi: 10.1177/20543581211018528 (PMC8182215; doi:10.1177/20543581211018528)
Supplement: sj-pdf-1-cjk-10.1177_20543581211018528 – Supplemental material for Cost of Potentially Preventable Hospitalizations Among Adults With Chronic Kidney Disease: A Population-Based Cohort Study [file sj-pdf-1-cjk-10.1177_20543581211018528.pdf]

Appendix 1. Number and percentage of encounters for individual CIHI-defined and CKD-related ACSC conditions

		Chronic Kidney Disease Risk Category						
		Low (n=91,126)	Moderate (n=16,413)	High (n=8,124)	Very high (n=6,596)	Dialysis (n=927)	High with unmeasured proteinuria (n=1,600)	Overall (n=124,786)*
CIHI-defined ACSC	# of ACSC encounters	4,993	2,057	1,744	2,341	202	563	11,900
	Percent of all ACSC encounters	42.0	17.3	14.7	19.7	1.7	4.7	100.0
	Grand mal and other epileptic convulsions	303	53	18	18	10	3	405
	% of all Grand mal encounters	74.8	13.1	4.4	4.4	2.5	0.7	100.0
	Coronary obstructive pulmonary disease (COPD)	2,377	793	567	513	46	194	4,490
	% of all COPD encounters	52.9	17.7	12.6	11.4	1.0	4.3	100.0
	Asthma	196	33	11	4	1	2	247
	% of all Asthma encounters	79.4	13.4	4.5	1.6	0.4	0.8	100.0
	Diabetes	622	246	147	206	51	10	1,282
	% of all Diabetes encounters	48.5	19.2	11.5	16.1	4.0	0.8	100.0
	Heart failure (HF) and pulmonary edema (PE)	901	723	848	1,408	67	310	4,257
	% of all HF/PE encounters	21.2	17.0	19.9	33.1	1.6	7.3	100.0
	Hypertension	159	81	65	102	20	16	443
	% of all Hypertension encounters	35.9	18.3	14.7	23.0	4.5	3.6	100.0
	Angina	435	128	88	90	7	28	776
	% of all Angina encounters	56.1	16.5	11.3	11.6	0.9	3.6	100.0
CKD-related ACSC	# of ACSC encounters	1,537	994	1,049	1,734	175	371	5,860
	Percent of all ACSC encounters	26.2	17.0	17.9	29.6	3.0	6.3	100.0
	Diabetes with ketoacidosis	358	123	63	66	39	4	653
	% of all Diabetes with ketoacidosis encounters	54.8	18.8	9.6	10.1	6.0	0.6	100.0
	Diabetes with hyperosmolarity	41	20	15	11	0	1	88
	% of all Diabetes with hyperosmolarity encounters	46.6	22.7	17.0	12.5	0.0	1.1	100.0
	Volume overload	6	4	5	31	35	3	84
	% of all Volume overload encounters	7.1	4.8	6.0	36.9	41.7	3.6	100.0
	Hyperkalemia	14	14	17	86	33	10	174
	% of all Hyperkalemia encounters	8.0	8.0	9.8	49.4	19.0	5.7	100.0
	Malignant hypertension	12	4	8	4	0	1	29
	% of all Malignant hypertension encounters	41.4	13.8	27.6	13.8	0.0	3.4	100.0
	Heart failure	1,106	829	941	1,536	68	352	4,832
	% of all Heart failure encounters	22.9	17.2	19.5	31.8	1.4	7.3	100.0

Abbreviations: ACSC: ambulatory care sensitive condition, CIHI: Canadian Institute for Health Information, CKD: chronic kidney disease.

* Among individuals with at least one hospitalization within the study period.
